# Highly-stretchable 3D-architected Mechanical Metamaterials

**DOI:** 10.1038/srep34147

**Published:** 2016-09-26

**Authors:** Yanhui Jiang, Qiming Wang

**Affiliations:** 1 Sonny Astani Department of Civil and Environmental Engineering, University of Southern California, Los Angeles, CA 90089, USA

## Abstract

Soft materials featuring both 3D free-form architectures and high stretchability are highly desirable for a number of engineering applications ranging from cushion modulators, soft robots to stretchable electronics; however, both the manufacturing and fundamental mechanics are largely elusive. Here, we overcome the manufacturing difficulties and report a class of mechanical metamaterials that not only features 3D free-form lattice architectures but also poses ultrahigh reversible stretchability (strain > 414%), 4 times higher than that of the existing counterparts with the similar complexity of 3D architectures. The microarchitected metamaterials, made of highly stretchable elastomers, are realized through an additive manufacturing technique, projection microstereolithography, and its postprocessing. With the fabricated metamaterials, we reveal their exotic mechanical behaviors: Under large-strain tension, their moduli follow a linear scaling relationship with their densities regardless of architecture types, in sharp contrast to the architecture-dependent modulus power-law of the existing engineering materials; under large-strain compression, they present tunable negative-stiffness that enables ultrahigh energy absorption efficiencies. To harness their extraordinary stretchability and microstructures, we demonstrate that the metamaterials open a number of application avenues in lightweight and flexible structure connectors, ultraefficient dampers, 3D meshed rehabilitation structures and stretchable electronics with designed 3D anisotropic conductivity.

Soft materials with a unique combination of tailored 3D architectures, lightweight and high deformability are desirable for a diverse range of scientific and technological applications including impact absorbents^1^,^2^, switchable acoustic modulators^3^,^4^, soft robotics^5^,^6^,^7^,^8^, rehabilitation devices^9^, stretchable electronics^10^, tissue scaffolds^11^ and drug delivery vehicles^12^. Towards manufacturing these soft materials, various strategies have been proposed, such as voiding elastomers with porogens^12^,^13^, directly writing elastomer/hydrogel inks^2^,^14^,^15^,^16^, casting hydrogels around perfusable structures^17^,^18^, and lithography-assisted etching of silicone elastomers^10^. Although these exiting paradigms have made tremendous progress, the created architected soft materials still suffer from limitations in either small stretchability or 3D architecture choices (such as stochastic foams^1^,^13^ and orthogonally layered beams^14^,^19^). Elastomer lattices with nanoarchitectures fabricated by an inverse processes of photolithography can be stretched to ~225%^10^; however, the lattices are limited to selective architectures that are determined by the waveguide etching pathways. A general approach to manufacture architected soft materials with integrated features of arbitrary 3D micro to miliarchitectures and reversible stretchability larger than 225% is still lacking^10^,^20^. Due to the manufacturing inability, from the fundamental perspective, a general and systematic understanding how the 3D architectures affect the mechanical efficiencies of stretchable soft materials remains primarily elusive^1^,^21^; and from the application perspective, a number of appealing applications that require both 3D complex architectures and high stretchability are still challenging, such as soft robots with low density (high porosity), tailored stiffness gradient and ultrahigh deformability^22^, or stretchable electronics with low density and prescribed 3D anisotropic conductivity^10^,^23^.

From another aspect, aided with modern additive manufacturing techniques (e.g., stereolithography and two-photon lithography), engineers have recently demonstrated potent capability of creating exotic rigid metamaterials in highly-complex 3D micro/nano architectures to enable ultralow material density and ultrahigh mechanical efficiency^24^,^25^,^26^,^27^,^28^,^29^,^30^,^31^. These additive manufacturing techniques, if successfully implemented to create highly-stretchable architected soft materials, would not only enable nearly arbitrary 3D architecture choices, but also significantly enhance architecture-assisted mechanical efficiencies or bring new functions of the existing soft materials. Despite the potential, limited progress has been made due to outstanding roadblocks on the way of direct additive manufacturing of highly-stretchable 3D-architected soft materials, including high viscosity, long curing time and self-weight supporting of elastomer/hydrogel monomers^20^.

Here, we propose a simple manufacturing strategy and create a class of mechanical metamaterials in nearly arbitrary 3D architectures of highly-stretchable elastomer lattices. The manufacturing strategy relies on dissolvable hollow scaffolds fabricated by projection microstereolithography and judiciously circumvents the existing roadblocks of additive manufacturing of elastomer microstructures^20^. The manufactured metamaterials are not only highly architected to enable density as low as 60 kg/m^3^ (6% of bulk elastomer), but also ultra-stretchable with reversible strain as large as 414%, 4 times larger than that of the existing counterparts with the similar complexity of 3D architectures^32^. With the unprecedented capability of sorting out bending or stretching dominant 3D architecture types, we are able to reveal their exotic mechanical properties: Under large-strain tension, metamaterial moduli are linear to their densities regardless of structural connectivity, different from the architecture-dependent modulus power-law of the existing engineering materials. Under large-strain compression, the metamaterials exhibit programmable negative stiffness which leads to ultrahigh energy absorption efficiencies. By integrating ultrahigh stretchability and designable architectures, the new metamaterials open promising avenues for not only reaching a new property spaces^1^,^33^, but also enable a variety of unprecedented applications on the human-machine and human-environment interfaces^20^. To demonstrate the potential, we show that the metamaterials can be tailored into customized geometries for lightweight connectors to flexibly bridge various 3D printed components, highly-efficient dampers that are better than the existing elastomer foams, rehabilitation structures that can conformally support organs with prescribed rigidity distribution and lightweight stretchable electronics with designed 3D conductivity pathways.

## Results

### Fabrications

**Figure 1:**
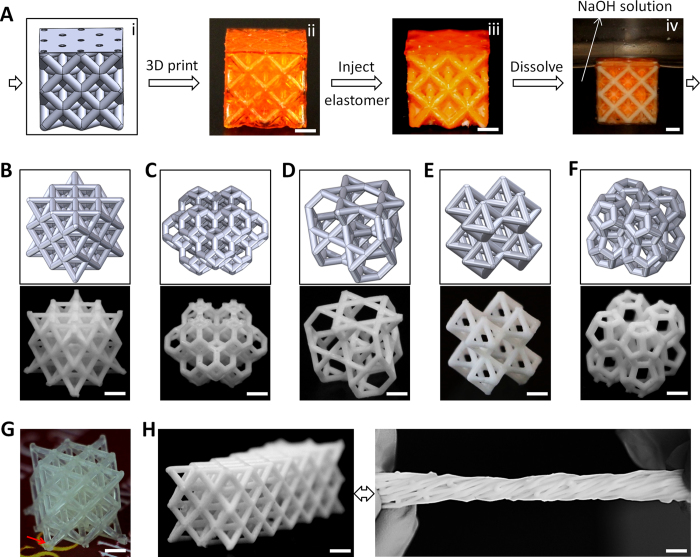
Fabrications of elastomer lattices. (**A**) Fabrication process of an elastomer lattice: A designed CAD model (i) is 3D-printed into a hollow scaffold (ii). The liquid prepolymer is then filled into and cured within the hollow channels of the scaffold (iii), which is subsequently dissolved in NaOH solution (iv). (**B**–**F**) CAD designs and fabricated elastomer lattices in various architectures: (**B**) Octet-truss, (**C**) Kelvin, (**D**) Kagome, (**E**) Octahedron and (**F**) Dodecahedron. (**G**) A transparent Octet elastomer lattice made of the transparent Mold max T10 elastomer. The arrow indicates the transparence of the beam. (**H**) An Octet elastomer lattice is reversibly twisted. All scale bars denote 2 mm.

The elastomer metamaterials are fabricated via a postprocessing of the projection microstereolithography^26^, in analogous to the fabrication of hollow metallic microlattices with microstereolithography^26^,^34^, waveguide polymerization^25^,^27^ or two-photon lithography^24^,^28^,^29^,^30^,^31^. To fabricate hollow metallic microlattices, polymer microlattices with solid beams are first fabricated, followed by a post-deposition of metals around the contour surfaces of the microlattices and a subsequent thermal decomposition of the polymer cores^26^,^27^,^28^,^30^. Here, we invert the process to first additively manufacture a highly-architected hollow polymer microlattice scaffold, then cure elastomers within the hollow channels, and finally chemically dissolve the hollow scaffold^19^,^35^, thus leaving a freestanding elastomer lattice (Fig. 1A, Supplementary Figs 1 and 2, see Materials and Methods). This facile manufacturing strategy is advantageous in circumventing the outstanding difficulties including high viscosity, long curing time and self-weight supporting of stretchable-elastomer monomers, which widely exist among the extrusion-based and photopolymerization-based additive manufacturing of elastomers^2^,^14^,^16^,^20^. The method enables us to fabricate a variety of elastomer lattices with nearly arbitrary 3D architectures, such as Octet-truss (Fig. 1B), Kelvin (Fig. 1C), Kagome (Fig. 1D), Octahedron (Fig. 1E) and Dodecahedron lattices (Fig. 1F), which are orderly assembled from a number of corresponding unit cells (Supplementary Fig. 3)^26^,^30^,^36^. The resolutions of the fabricated elastomer lattices are determined by the diameters of the hollow channels, in a range from 100 to 1500 μm. The manufacturing method is very general, applicable for a number of stretchable elastomer materials, such as tin-catalyzed silicones and urethane elastomers, either opaque or transparent (semi-transparent) (Fig. 1B–G, Supplementary Fig. 4). Due to the high stretchability of the elastomer constituents, the fabricated lattices are highly deformable to reversibly sustain large-strain compression, tension and torsion (Fig. 1H, Supplementary Fig. 5). In addition, the fabricated lattices are very lightweight, with effective densities in a range of 60–500 kg/m^3^ that are around 6–50% of the corresponding elastomer bulk. Since the elastomer modulus is in a range of 20–400 kPa (Supplementary Fig. 6), once the relative density is lower than 6% (e.g., beam diameter smaller than 40 μm), achieving freestanding elastomer lattices is challenging because the surface tension plays a significant role in deforming the elastomer lattices into crumpled geometries^37^. Furthermore, the method is scalable because the elastomer lattices can be easily scaled up to large areas or volumes (e.g., ~1000 cm^3^) by increasing the volume of the hollow scaffolds and the number of unit cells with the existing stereolithography technique combining x-y axis scanning (Fig. 1H)^26^.

### Comparison with existing engineering lattices

**Figure 2:**
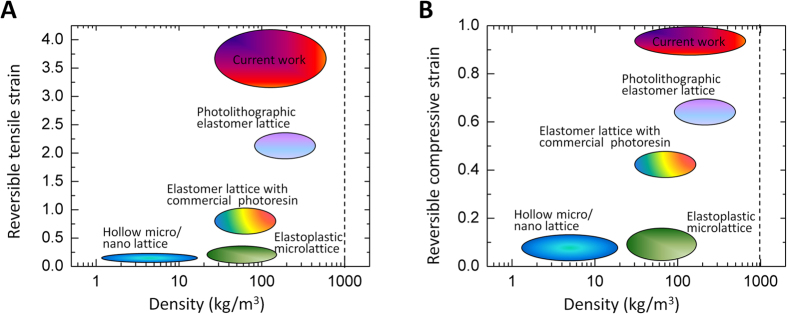
Property comparison with existing architected materials. Comparison of the maximum reversible (**A**) tensile strain and (**B**) compressive strain between the current elastomer lattices and other existing lattice materials with 3D complex architectures as functions of their densities. The dash lines represent the density of the elastomer solid.

**Figure 3:**
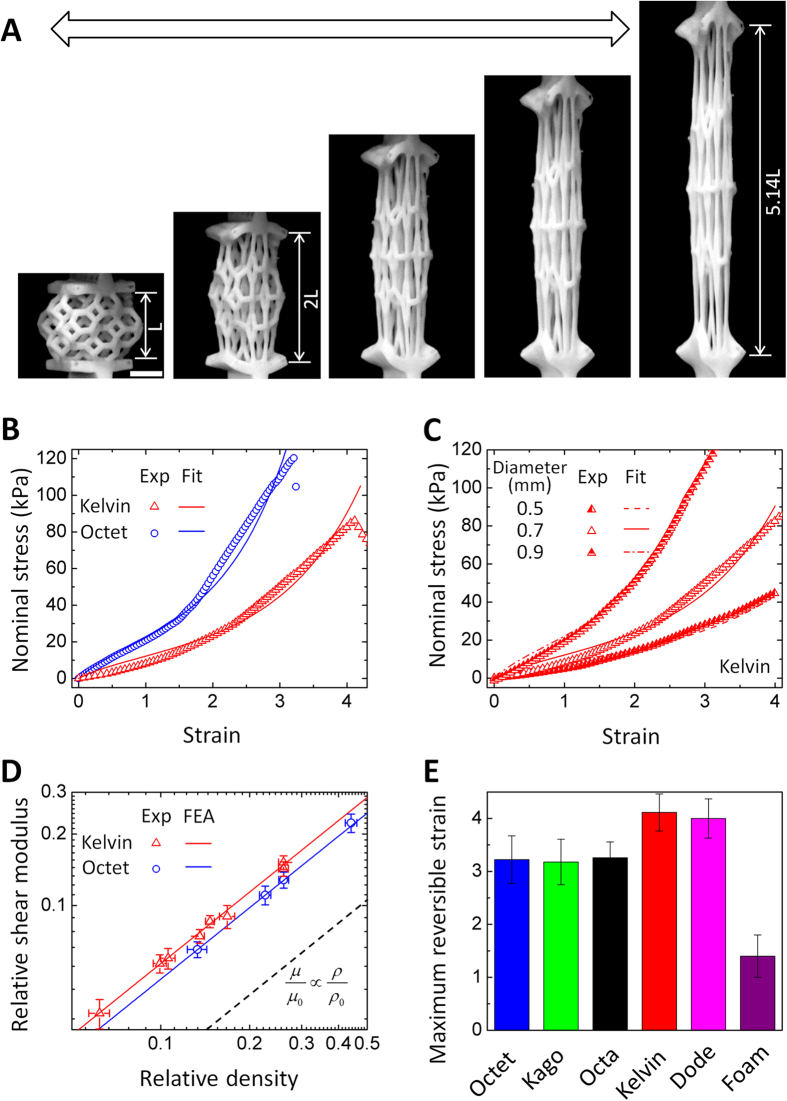
Mechanical behaviors of elastomer lattices under large-strain tensions. (**A**) Sequential images to show a Kelvin elastomer lattice under increasing uniaxial tensile strains. The arrow indicates the reversibility of the stretching. Scar bar denotes 4 mm. (**B**) Nominal stresses of Kelvin and Octet elastomer lattices in functions of tensile strains. The experimentally measured curves (“Exp”) are fitted to the Arruda-Boyce model with μ = 6 kPa and λ_m_ = 2.8 for Kelvin, and μ = 10.1 kPa and λ_m_ = 2.3 for Octet. (**C**) Nominal stresses of Kelvin elastomer lattices with varied beam diameters in functions of tensile strains. The fitted shear moduli are 4.2 kPa (0.5 mm), 6 kPa (0.7 mm) and 10 kPa (0.9 mm), respectively. (**D**) Relative shear moduli of Kelvin and Octet elastomer lattices in functions of relative densities. The finite-element-analysis (“FEA”) simulated relationships are μ/μ_0_ ≈ 0.57 ρ/ρ_0_ (Kelvin) and μ/μ_0_ ≈ 0.49 ρ/ρ_0_ (Octet). The dash line in (**D**) indicates the slope for scaling relationship μ/μ_0_ ∝ ρ/ρ_0_. (**E**) The maximum reversible tensile strains of Octet, Kagome, Octahedron, Kelvin and Dodecahedron elastomer lattices, and elastomer foams. The error bars denote the standard deviation among at least 3 data points.

Compared to reported architected materials with 3D complex architectures (not including foams with stochastic architectures), our elastomer lattices pose the highest reversible stretchability and compressibility (Fig. 2). The fabricated elastomer-lattice metamaterials can be reversibly stretched over 414% strain (Fig. 3A) and compressed over 98% strain (Supplementary Fig. 5). However, the solid or hollow micro/nano lattices (including rigid plastics, metals and ceramics) fabricated by projection microstereolithography^25^,^26^,^34^, waveguide polymerization^27^ and two-photon lithography^24^,^28^,^29^,^30^,^31^ generally can only be reversibly compressed or stretched up to ~15% strain before yielding or fracture. Elastomer lattices fabricated with the commercially-available photoresins (usually thermoplastic elastomer) via stereolithography can only be reversibly compressed to ~50% and stretched to ~80%^5^,^38^. Elastomer nanolattices fabricated by an inverse processes of photolithography can be reversibly compressed to ~70% and stretched to ~225%^10^; however, they are limited to selective architectures that are determined by the waveguide etching pathways. As a result, the reversible stretchability of the elastomer metamaterials presented in the current paper (414% strain) is more than four times larger than that of lattice structures in similar level of architecture complexity (e.g., stereolithography of commercial thermoplastic elastomer). In addition to the outstanding deformability, our elastomer metamaterials are also very lightweight: Compared to the elastomer solids with density ~1000 kg/m^3^ (shown as the dash lines in Fig. 2), the density of our elastomer lattices are only 6–50% of their bulk, in a comparable range of those of the microlattices fabricated by the existing additive manufacturing techniques (Fig. 2)^25^,^26^,^27^,^34^.

### Large-strain tension

It is well known that the small-strain Young’s moduli of lattice materials scale with their relative densities in power laws as^1^


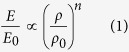


where *E* and *E*_o_, *ρ* and *ρ*_o_ are Young’s moduli, density of the lattices and the solid constituents, respectively. The exponent *n* is strongly dependent on the architectures^1^,^21^,^36^: n ~ 2–3 if the constituents carry loads primarily in bending, and n ~ 1 if the loaded constituents mainly under tension or compression. The former is called *bending-dominant structure*, with examples including Octet-truss (Fig. 1B) and Octahedron (Fig. 1E); and the latter *stretching-dominant structure*, with examples including Kelvin (Fig. 1C) and Dodecahedron (Fig. 1F). Although the modulus law for small strains (within 10%) is well characterized^1^,^21^,^36^, the modulus law for highly-architected structures under large-strain (e.g., >100%) remains unclear.

To probe large-strain tensile behaviors of elastomer lattices, we apply a quasistatic stretching (loading rate 0.0167 mm/s) to the fabricated lattices (Fig. 3A, sample fabrication in Methods and Supplementary Fig. 2B). We find that the elastomer lattices can reversibly sustain 414% uniaxial strain before the first beam fracture (Fig. 3A, Supplementary Movie 1). Under uniaxial stretch, lattice beams are first aligned along the stretching direction, and aligned beams are then stretched to larger strains until fracture (Fig. 3A, Supplementary Movie 1). As the structures are under large tensile strain (e.g., strain > 100% in Fig. 3A), most of the lattice beams are either stretched or compressed, regardless of stretching or bending dominant architectures (Fig. 3A, finite element analyses in Supplementary Figs 6 and 7). Therefore, we hypothesize that both stretching and bending-dominant structures would follow a stretching-dominant behavior under large-strain tensions. To test this hypothesis, we first calculate the large-strain effective shear moduli of elastomer lattices by fitting the experimentally measured stress-strain curves to a hyperelastic constitutive model, Arruda-Boyce model, which gives uniaxial nominal stress as^39^ (Fig. 3B)


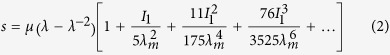


where *μ* is shear modulus, *λ* = 1 + *ε* is uniaxial stretch, *I*_1_ = *λ*^2^ + 2*λ*^−1^ is the strain invariant and *λ*_*m*_ is a parameter to characterize the maximum stretchability. Then, we systematically vary lattice densities by changing lattice beam diameters, which lead to variations of their shear moduli (Fig. 3C). As we plot out the shear moduli as functions of lattice relative densities *ρ*/*ρ*_o_ (Fig. 3D), we find that the shear moduli of elastomer lattices in both stretching and bending-dominant architectures follow a linear scaling with their relative densities (e.g., Octet and Kelvin in Fig. 3D), i.e.,


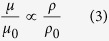


where *μ* and *μ*_0_ are shear moduli of the lattices and the constituent beams, respectively. For stretching-dominant Octet lattices, Equation 3 is well consistent with the scaling law for small-strain modulus behaviors (Equation 1). However, the linear modulus law for the bending-dominant Kelvin is in sharp contrast to the square or cubic scaling law for small-strain modulus of existing bending-dominant lattices (Equation 1)^1^,^36^. The linear scaling for the Kelvin lattices is because that they, like the stretching-dominant lattices, also primarily stretch or compress their beams during the large-strain stretching (strain > 100%) (Fig. 3A, Supplementary Fig. 7). This deformation mechanism is consistent with those of highly-deformable structured proteins^40^ and soft composites^41^. To further understand and numerically verify the scaling relationship shown in Eq. 3, we implement finite element computations whose results show that μ/μ_0_ ≈ 0.49 ρ/ρ_0_ for Octet elastomer lattice, and μ/μ_0_ ≈ 0.57 ρ/ρ_0_ for Kelvin, both consistent with the experiments (Fig. 3D, Supplementary Figs 7 and 8). Moreover, experiments and computations on Octahedron and Dodecahedron lattices further verify that Eq. 3 is a general scaling law for large-strain moduli of both stretching and bending-dominant elastomer lattices (Supplementary Figs 8 and 9).

Under ultra-large tensile strains, the elastomer lattices fracture their beams, most likely initiated from defects on the lattice beams (Supplementary Fig. 10, Supplementary Movie 1). The experiments show that the fabricated elastomer lattices can be reversibly stretched up to 320–414% strains until the first beam-fracture (Fig. 3E). Before the first beam-fracture, the elastomer lattices can be reversibly stretched for more than 100 cycles with negligible hysteresis after the first loading-unloading cycle (Supplementary Fig. 11, Supplementary Movie 1). It is noted that the reversible stretchability of the current elastomer lattices (strain 320–414%) is much larger than that of elastomer foams fabricated with the same elastomer materials (strain ~ 100–170%) (Fig. 3E). It is probably because elastomer foams in a stochastic architecture usually embed some irregular ultra-thin beams that can be easily fractured at a relatively small force, and the initiated cracks generate stress concentrations that may further promote the crack propagation (Supplementary Fig. 12).

### Large-strain compression

**Figure 4:**
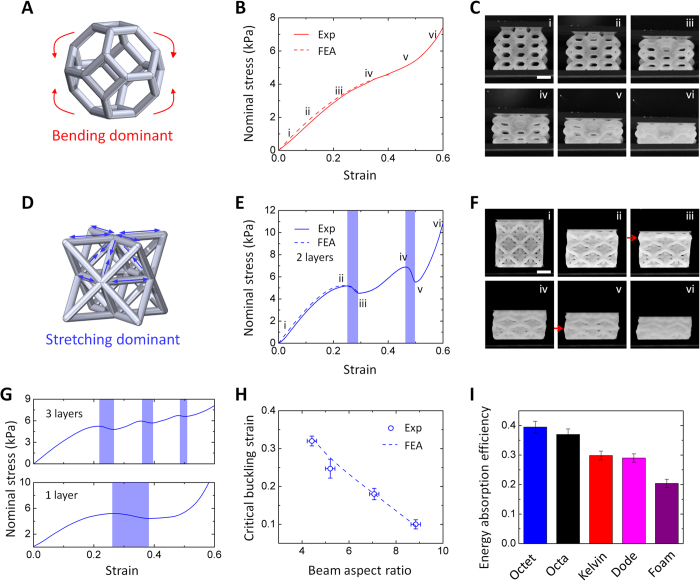
Mechanical behaviors of elastomer lattices under large-strain compressions. (**A**) The mechanical response to compressive loading on a Kelvin unit cell. (**B**) Nominal stresses of a Kelvin elastomer lattice in a function of compressive strains with loading rate 0.0167 mm/s. (**C**) Sequential images (i–vi) of the Kelvin lattice under large-strain compression denoted in (**B**). (**D**) The mechanical response to compressive loading on an Octet unit cell. (**E**) Nominal stresses of an Octet elastomer lattice (2 × 2 × 2) in a function of compressive strains with loading rate 0.0167 mm/s. (**F**) Sequential images (i–vi) of the Octet lattice under large-strain compression denoted in (**E**). The red arrows in (**F**) denote the buckling layers in the Octet lattice. (**G**) Nominal stresses of 2 × 2 × 1 and 2 × 2 × 3 Octet elastomer lattices in functions of compressive strains with loading rate 0.0167 mm/s. Shadow regions in (**E**) and (**G**) indicate the negative stiff regions. (**H**) The critical strains of the first buckling of the Octet lattices in a function of the beam aspect ratios (beam length/diameter). (**I**) Energy absorption efficiencies of Octet, Octahedron, Kelvin and Dodecahedron elastomer lattices, and an elastomer foam, whose relative densities are all ~26%. The error bars denote the standard deviation among at least 3 data points. Scale bars in (**C**,**F**) denote 2 mm.

Unlike the large-strain tensile behaviors, we find that the compressive behaviors of bending and stretching dominant lattices are remarkably different. For a bending-dominant Kelvin elastomer lattice, the stress response primarily originates from the large-deflection bending of the elastomer beams (Fig. 4A), and thus increases monotonically with the applied strain (Fig. 4B,C, Supplementary Movie 2)^42^. This observation is similar to the compressive behaviors of the elastomer foams with stochastic bending-dominant architectures^1^,^42^. Differently, the compressive loading on a stretching-dominant Octet elastomer lattice is primarily applied along the beam axes (Fig. 4D), and can trigger beam buckling after a critical load. Therefore, the stress response of the Octet lattice is non-monotonic, but feature domains with negative stiffness after the occurrence of beam buckling (Fig. 4E,F, Supplementary Movie 3). It is noted that these different compressive behaviors are not limited to Kelvin and Octet lattices, but also occur in other bending-dominant (e.g., Dodecahedron) and stretching-dominant (e.g., Octahedron) lattices (Supplementary Fig. 13). To understand the distinct behaviors, we perform finite element analyses for Octet and Kelvin lattices under large-strain compressions. The computational results show that the stress response of the compressed Kelvin lattice indeed monotonically increases with the applied strain (Supplementary Fig. 14), while the stress response of the compressed Octet lattice goes up and down due to the beam buckling (Supplementary Fig. 15). The computational predictions quantitatively agree with the experimentally measured stress-strain curves (Fig. 4B,E).

It is observed that for an Octet lattice with two layers of unit cells, the buckling initiates at one cell layer of Octet lattices, and increasing compressions can further trigger the subsequent buckling at the second layer of cells (Fig. 4E,F)^43^. Based on this observation, we can further tune the domain number and strain range of the buckling-induced negative stiffness (Fig. 4E,G): Lattices with one layer of Octet cells feature one domain with negative stiffness within strain 0.26–0.37, while lattices with two and three layers of Octet cells feature two and three domains with negative stiffness, respectively. Moreover, the initial strain of the negative stiffness can also be tuned by varying the aspect ratios of the lattice beams: the critical buckling strains of Octet lattices monotonically decrease with increasing beam aspect ratios, consistent with the finite element calculations (Fig. 4H).

The sequential buckling on the stretching-dominant lattices can maintain the stress-level throughout a long plateau region (Fig. 4E,G), thus facilitating the lattices to absorb a large amount of elastic energy without dramatically increasing the stress^2^,^44^. To indicate the energy absorption capability, a parameter called energy absorption efficiency can be defined as stored elastic energy in the lattice normalized by maximum applied stress^1^,^44^,^45^ (calculations in Supplementary Materials and Supplementary Fig. 16). We find that due to the sequential buckling the energy absorption efficiencies of the stretching-dominant lattices (e.g., Octet and Octahedron) under quasistatic loading (rate 0.0167 mm/s) are higher than those of the bending-dominant lattices (e.g., Kelvin and Dodecahedron), given the same lattice densities (ρ/ρ_0_ ~ 26% in Fig. 4I, ρ/ρ_0_ ~ 14% in Supplementary Fig. 17). The behaviors are also consistent for various high loading rates (e.g., 0.83 and 3.33 mm/s in Supplementary Fig. 18). In addition, because the stochastic architectures of elastomer foams usually follow bending-dominant behaviors^12^,^36^,^37^, elastomer foams’ energy absorption efficiency is much lower than those of stretching-dominant elastomer lattices (Fig. 4I, Supplementary Figs 17–19). Furthermore, unlike the existing elastoplastic lattices (e.g., rigid plastic, metal or ceramic) that can only be used for energy absorption once due to the yielding or fracture^1^,^44^,^45^, our elastomer lattices can reversibly absorb energy for more than 100 cycles (Supplementary Figs 11 and 20, Supplementary Movies 2 and 3).

## Discussion

**Figure 5:**
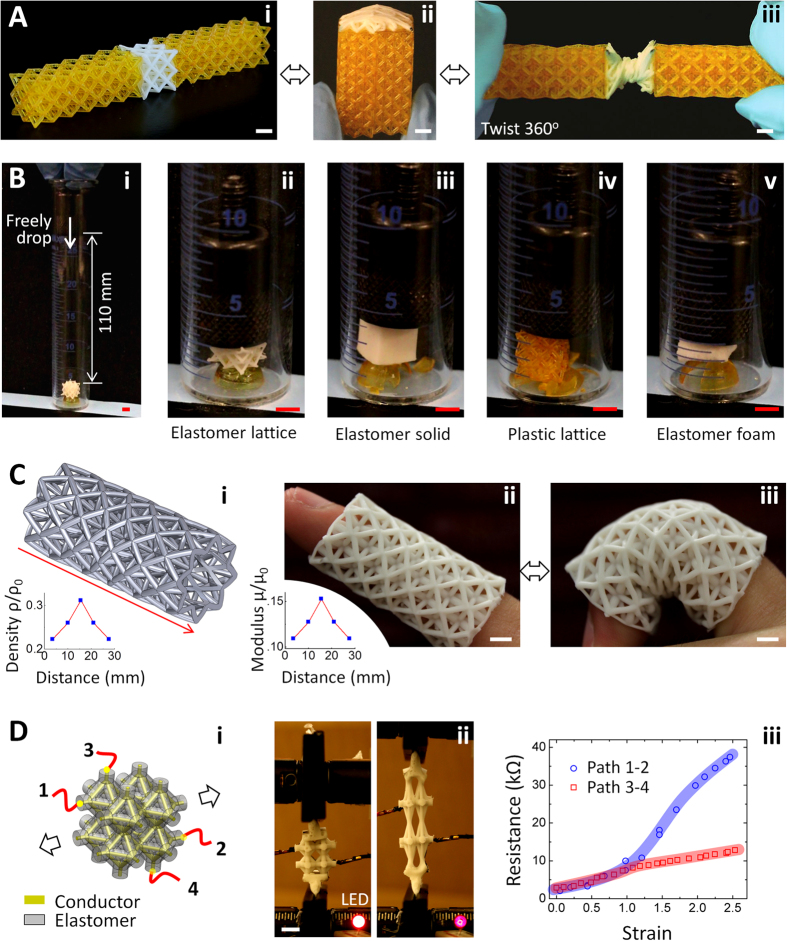
Applications of elastomer-lattice structures. (**A**) An Octet elastomer lattice bonded between two Octet HDDA lattices (i) can sustain bending by 180° (ii) and twisting by 360°. (**B**) A metal weight (36 g) freely dropped from 100 mm height onto a damper-protected HDDA thin shell (i). The HDDA shell is still intact if the damper is an Octet elastomer lattice (ii, ρ/ρ_0_ = 26%), but all broken if the damper is an elastomer solid (iii), an Octet HDDA lattice (iv, ρ/ρ_0_ ~ 26%) or an elastomer foam (v, ρ/ρ_0_ = 26%). (**C**) A finger rehabilitation device: (i) A cylindrical lattice with gradient density along the longitudinal direction (marked by the red arrow). The inset shows the calculated relative density along the longitudinal path. (ii) A fabricated elastomer lattice sample with gradient shear modulus along the longitudinal path (shown in the inset). The shear modulus is calculated by μ/μ_0_ = 0.49 ρ/ρ_0_ (Octet). (iii) The finger covered with the elastomer lattice can reversibly undergo large-strain bending. (**D**) A stretchable elastomer-lattice conductor: (i) A schematic to show the stretchable conductor structure. 1–4 denotes the wire connectors that can be connected to the electric circuit shown in Supplementary Fig. 25. (ii) The stretchable conductor under tensile strain ~2.5 is still conductive enough to power a LED light. (iii) The resistances along different conductive paths (e.g., path 1–2 and path 3–4) in functions of increasing tensile strains. All scale bars denote 4 mm.

Thanks to the unprecedented combination of high-stretchability, high-complexity in architecture, low density and easy-shapeability, the elastomer metamaterials can facilitate a number of applications. First, in robotic and 4D printing applications, connectors are required to bridge parts to flexibly bend or rotate; however, the existing connectors are either thermoplastics with drawbacks in limited deformability and cyclability, or bulk soft materials short in high density^8^,^32^,^46^,^47^,^48^. Here we demonstrate that our elastomer lattices can enable high-flexibility of the structural bridging between 3D printed rigid plastic parts to reversibly sustain large-strain stretching, compression, bending and twisting, yet keeping the whole system at a very low density (Fig. 5A, Supplementary Fig. 21).

Second, our elastomer lattices with ultrahigh energy absorption efficiencies can be used as extraordinary dampers to protect precision components or devices, with higher efficiency than existing elastomer foams, a well-known excellent damper^1^ (Figs 5B and 3I). To prove the concept, we fabricate identical plastic thin shells in a semi-spherical shape (thickness ~400 μm, strong enough to sustain 36 g weight shown in Supplementary Fig. 22), and then use an elastomer lattice (ρ/ρ_0_ ~ 26%, Fig. 5Bii), an elastomer solid (Fig. 5Biii), a plastic lattice (ρ/ρ_0_ ~ 26%, Fig. 5Biv) and an elastomer foam (ρ/ρ_0_ ~ 26%, Fig. 5Bv) as dampers to shield impacts. Repeated experiments show that under impacts of freely-dropping of identical weights (36 g) from 100 mm height, the shell protected by the elastomer lattice is still intact, while others all broken (Fig. 5B, Supplementary Fig. 22, Supplementary Movie 4). The elastomer lattices can also be effective for cylindrical and cubic shells (Supplementary Fig. 23). Compared to the reported 2.5D elastomer dampers by Shan *et al*.^2^, the current elastomer lattices have high damping performance for three Cartesian coordinates. In addition, the current lattices can autonomously return to the original shapes once the external load is retrieved, different from the cases in Shan *et al*.^2^ which require external activations.

Third, our elastomer lattices in defined shapes can be used as disability rehabilitation devices that support the large-distance movement of organ joints during the rehabilitation practice^9^ (Fig. 5C). The existing rehabilitation devices are limited in high density and inability in designing customized supporting gradient^49^. Here we show a low-density (ρ/ρ_0_ ~ 26%) elastomer Octet lattice in a cylindrical shape can support a finger to perform bending practices (Fig. 5C, Supplementary Fig. 24, and Supplementary Movie 5). A prescribed gradient density is designed along the longitudinal direction, inducing gradient moduli to provide customized force-supports for specific parts along the finger, which may greatly facilitate the healing process of the finger (Fig. 5C, see homogeneous density case in Supplementary Fig. 24)^9^,^49^.

Finally, the elastomer lattices can also be used for flexible and wearable electronics (Fig. 5D). The existing flexible conductors are limited in either low stretchability, high density or inability to achieve 3D anisotropic conductivity through designed conductive paths^10^,^23^. With designed compliant conductive channels within the elastomer lattice beams (Fig. 5Di, Supplementary Fig. 25A), the elastomer lattices with low density 0.23 g/cm^3^ can remain conductive within cyclic uniaxial strain as large as 250% (Fig. 5Dii, Supplementary Fig. 25B, Supplementary Movie S6). The lattices also exhibit prescribed anisotropic conductivity during the large-strain stretching. For example, the conductivity along path 1–2 (along the stretching direction) and path 3–4 (normal to stretching direction) are different due to the different stretching ratio along the conductive paths (Fig. 5Biii).

It is noted that the stretchability of elastomer lattices in the current work has not been optimized. In the future study, the stretchability of elastomer lattices can be optimized through sorting out various elastomer parent materials with higher stretchability, or designing architectures to delay the deformation of the lattice beams^10^. It is also noted that platinum-catalyzed silicones (e.g., PDMS^10^) or hydrogels^14^ cannot be used in the current fabrication method, because platinum-catalyzed silicones cannot cure around the dissolvable scaffold, and the hydrogel would dissolve the scaffold during the filling step.

In summary, we present a class of mechanical metamaterials that features 3D highly-complex architectures and ultrahigh reversible stretchability (strain 414%). With full freedom of controlling architectures, the elastomer-lattice metamaterials with tunable exotic large-strain tensile and compressive properties may open new avenues for a number of future research directions and applications. In the research field of material science, these elastomer metamaterials demonstrate a new property space which features an unprecedented combination of 3D free-form architecture, low density and ultrahigh stretchability (Fig. 2)^1^; within this property space, these elastomer metamaterials can potentially mimic the hierarchical structures and mechanical performance of their natural counterparts (e.g., elastomer proteins) that have evolved for centuries^1^,^16^,^50^,^51^,^52^. These elastomer metamaterials may also pave ways for future designs of novel engineering materials with extreme stretchability and densities, which are specifically useful for unconventional electronics^23^, robotics^5^,^6^,^7^,^8^, exoskeleton devices^49^ and other bioinspired soft machines^53^,^54^. In addition, the elastomer metamaterials demonstrate reversible switch of 3D complex architectures under mechanical loadings, which may potentially facilitate dynamically interactions with various energy flows, such as shock impacts, acoustic waves and electromagnetic energies^2^,^3^,^55^, or show various abnormal material properties, such as negative indexes and cloaking^3^,^15^,^56^. Furthermore, coupled with stimuli-controlled actuations, the elastomer metamaterials with tailored geometries may show great potential in 4D printing to exhibit externally controlled changes of 3D shapes and associated functionalities^47^,^57^.

## Materials and Methods

### Fabrication of elastomer lattices and other structures

The fabrication of elastomer lattices starts from water-soluble hollow scaffolds that are 3D-printed by a projection microstereolithography system (Supplementary Fig. 1). The photoresin is a mixture of acrylic-based photopolymers N,N-Dimethylacrylamide (40% wt), Methacrylic acid (40% wt) and Methacrylic anhydride(7% wt), water soluble filler Polyvinylpyrrolidone (11% wt) and photoinitiator Phenylbis (2,4,6-trimethylbenzoyl) phosphine oxide (2% wt)^35^. First, a 3D hollow CAD model is sliced into a series of images with a prescribed spacing along the vertical direction. These 2D slice images, illuminated with UV/blue light from a light emitting diode, are sequentially projected onto a transparent window, on which the photoresin is capped in a prescribed height by a printing glass stage. The exposed resin is solidified, forming a layer structure bonded onto the printing stage. To eliminate the adhesion between the solidified resin and the transparent window, an oxygen permeable membrane (Teflon fluoropolymer, CSHyde, USA) is attached on the window, inducing a thin layer (~5–20 μm) of oxygen-rich dead zone to quench the photopolymerization^34^. As the printing stage is lifted off, fresh resin can be delivered beneath the printing stage by a rotational wheel. By lowering down the stage by a prescribed height and illuminating the resin with another slice image, a second layer can be printed and bonded onto the first layer. By repeating these processes, we can print a hollow scaffold with nearly arbitrary 3D architectures. Once dried with air for 2 h, the hollow scaffold is filled with tin-catalyzed silicone elastomers (mold max NV14 and 10T, Smooth-on, USA) and Urethane elastomers (PMC-724, Smooth-on, USA) with mixtures of the base and the crosslinker 10:1 and 1:1 by weight, respectively. The filling process is well controlled by a syringe equipped with a syringe pump, thus smoothly squeezing the air away from the outlets. The filled elastomers are cured for 12 h at 25 °C. Thereafter, the composites with scaffolds and cured elastomers are immerged in 1 mol/L NaOH solution for 6 h. The elastomers lattices are ready for use after washing in DI water for 2 min and air-drying for 2 min.

### Mechanical tests of elastomer lattices

In compression tests, elastomer lattices are compressed by various loading rates 0.0167, 0.83 and 3.33 mm/s with a mechanical tester (model 5942, Instron, USA). The nominal stress is calculated as the loading force over the average crosssection area of the lattice. The compressive strain is directly calculated as the loading distance over the initial height of the lattice. In tensile tests, the samples are clamped on the designed stretching bars and stretched by loading rate 0.0167 mm/s. The deformation evolution is recorded by a camera (Canon EOS 70D) and the tensile strain is calculated by the real elongating length of the lattice part (Fig. 2A).

### Calculation of relative densities of elastomer lattices

The beam diameter and length of the fabricated elastomer lattices are first measured with a vernier caliper (resolution 10 μm). Then a SolidWorks model is created with the measured dimensions. The relative density ρ/ρ_0_ is calculated as the volume fraction of the lattice structures, directly estimated in the SolidWorks model.

### Fabrication and mechanical tests of elastomer foams

The elastomer foams are fabricated by curing a mixture of mold max NV14 and sugar powder (size 100–500 μm, C&H sugar, USA) in a glass vial. The cured sample is then immerged in DI water for 6 h to dissolve the sugar, leaving a porous elastomer foam. A foam (cut in a disk with height 8 mm and diameter 8 mm) is compressed with loading rate 0.0167, 0.83 and 3.33 mm/s, respectively (Supplementary Figs 17–19). Foam bars with diameter 8 mm and length 35 mm are stretched with rate 0.0167 mm/s (Supplementary Fig. 12).

### Finite element analyses

All models are first designed in SolidWorks and then imported into a finite-element code, ABAQUS 6.10.1. The mold max NV14 elastomer beams are taken to obey Arruda-Boyce model with parameters calculated from Supplementary Fig. 6, namely μ_0_ = 71 kPa and λ_m_ = 2.91 (see Eq. 2). The models are discretized by C3D8R elements and the result accuracy is ascertained through mesh refinement studies. The loadings in the simulations are displacement-controlled. The loading force is calculated by integrating the reaction force on one end surface.

### Flexible connector

The rigid plastic lattices in Fig. 5A are fabricated by printing a photoresin with a mixture of 1,6-Hexanediol diacrylate (HDDA, 98% wt) and photoinitiator Phenylbis (2,4,6-trimethylbenzoyl)phosphine oxide (2% wt). Then the elastomer lattice is firmly bonded between two plastic lattices with a very thin layer of super glue (~200 μm, Gorrila, USA). The HDDA-elastomer lattice composites are bended and twisted by hands.

### Damping experiments

Identical semi-spherical, cubic and cylindrical shells with thickness ~400 μm (Supplementary Figs 22 and 23) are first fabricated by printing photoresin HDDA with the photoinitiator. An Octet elastomer lattice (ρ/ρ_0_ ~ 26%), an elastomer solid cubic, an HDDA lattice (ρ/ρ_0_ ~ 26%) and an elastomer foam (ρ/ρ_0_ ~ 26%) with the same sample size (8 mm × 8 mm × 8 mm) are placed on the identical HDDA shells with a very thin layer of super glue. The metal weight (~36 g) cannot break the skull (~0.16 g) by its own weight (Supplementary Fig. 22B). Same weights are freely dropped from 110 mm height onto various samples. To prevent the weight jumping, a glass tube is used during the damping experiment.

### Experiments on the elastomer-lattice conductor

An Octahedron elastomer lattice with hollow beams is first fabricated using the model in Supplementary Fig. 25A. The hollow channels (diameter 0.4 mm) within the elastomer lattice are first filled in a compliant conductor, carbon grease (MG chemicals, USA) with a syringe. The carbon grease can flow conformally during the large-stretching of the elastomer lattice. Some openings designed on the elastomer lattice (Fig. 5Di) are connected to an electric circuit with a DC voltage (24V), electric switch and a LED (Supplementary Fig. 25B). The cyclic stretching of the elastomer lattice is controlled by Instran mechanical tester with rate 0.01 mm/s. The electric resistances of the elastomer-lattice conductor through different conductive paths are measured with a resistance meter (Klein tools, USA).

## Additional Information

**How to cite this article**: Jiang, Y. and Wang, Q. Highly-stretchable 3D-architected Mechanical Metamaterials. *Sci. Rep.* **6**, 34147; doi: 10.1038/srep34147 (2016).

## Supplementary Material

Supplementary Information

Supplementary Movie 1

Supplementary Movie 2

Supplementary Movie 3

Supplementary Movie 4

Supplementary Movie 5

Supplementary Movie 6
