# Supplementary material for: Highly-stretchable 3D-architected Mechanical Metamaterials
Source: Sci Rep. 2016 Sep 26;6:34147. doi: 10.1038/srep34147 (PMC5035992; doi:10.1038/srep34147)
Supplement: Supplementary Information [file srep34147-s1.pdf]

Supplementary Materials for

Highly-stretchable 3D-architected Mechanical Metamaterials

Yanhui Jiang[1], Qiming Wang[1]*

[1]Sonny Astani Department of Civil and Environmental Engineering, University of Southern California, Los Angeles, CA 90089, USA.

*To whom corresponding should be addressed. Email: qimingw@usc.edu

Supplementary Methods

Energy absorption of elastomer lattices. As shown in **Supplementary Fig. 16**, with increasing compressive strains, elastomer lattices undergo three stages: first a linear elastic region up to ~10% strain, and then a plateau region with stagnant or slowly increasing stress levels, followed by a densification region with a rapid increase of stress. The energy absorption efficiency of an elastomer lattice is defined as (shown in **Supplementary Fig. 16AC**) [1-3]

$$\eta = \frac{\int_0^{\varepsilon_D} s d\varepsilon}{s_{tr}} \tag{S1}$$

where, ε is the compressive strain, s is the nominal stress in a function of compression strain ε, ε_D is the densification strain that denotes a initial strain for the densification stage, and $s_{tr} = \max[s(0 \rightarrow \varepsilon_D)]$ is the maximum transmitted stress.

In the practical calculations, the densification strain ε_D is unknown; therefore, we first sort out the energy absorption efficiency through increasing applied strain ε. i.e.,

$$\eta_1 = \frac{\int_0^{\varepsilon} s d\varepsilon}{s_{tr}} \tag{S2}$$

where $s_{tr} = \max[s(0 \rightarrow \varepsilon)]$ is the maximum transmitted stress. It is noted that s_{tr} is not necessarily equal to s (see a case in the inset of **Supplementary Fig. 16C**). Then, the energy absorption efficiencies

η shown in **Fig. 4I** are the maximum η_1 values across all applied strains (the maximum points in **Supplementary Fig. 16BD**). The densification strain is the strain corresponding to the maximum η_1 value (**Supplementary Fig. 16BD**). Calculation examples are shown in **Supplementary Figs. 13** and **16**.

Supplementary figures and captions

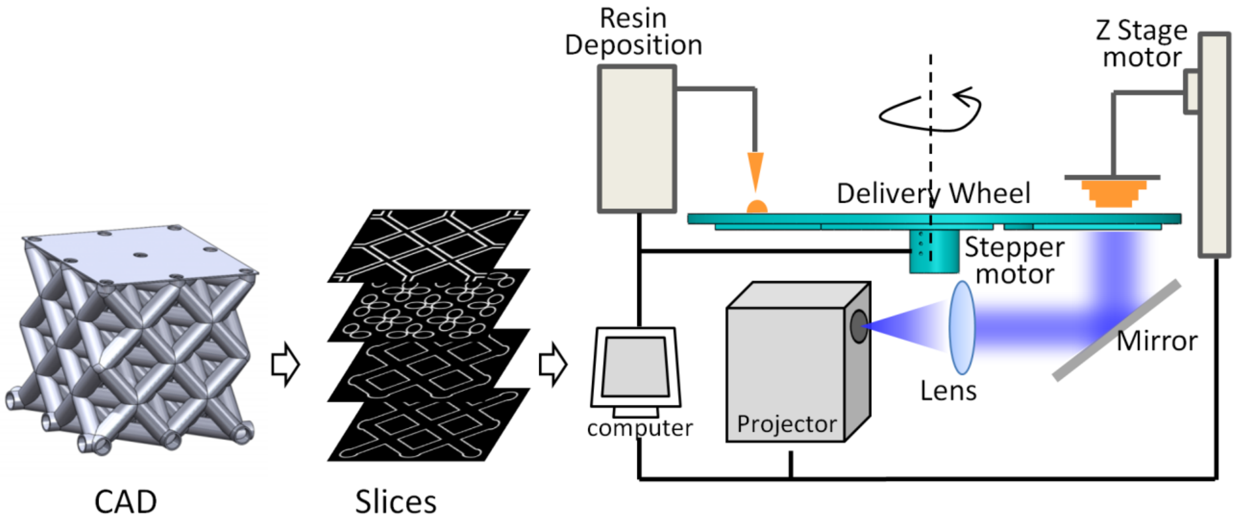

Supplementary Figure 1. 3D printing process via projection microstereolithography. A CAD model is first sliced into a sequence of images. These 2D slice images, illuminated with UV/blue light from a light emitting diode, are sequentially projected onto a transparent window. On the window, the liquid photoresin, capped into a prescribed height by a printing stage, is cured by the light and attached onto the printing stage. As the printing stage is lifted off, the fresh resin is delivered beneath the printing stage by a rotational wheel. By lowering down the stage by a prescribed height and illuminating the resin with a subsequent slice image, a new layer can be printed and bonded onto the former layer.

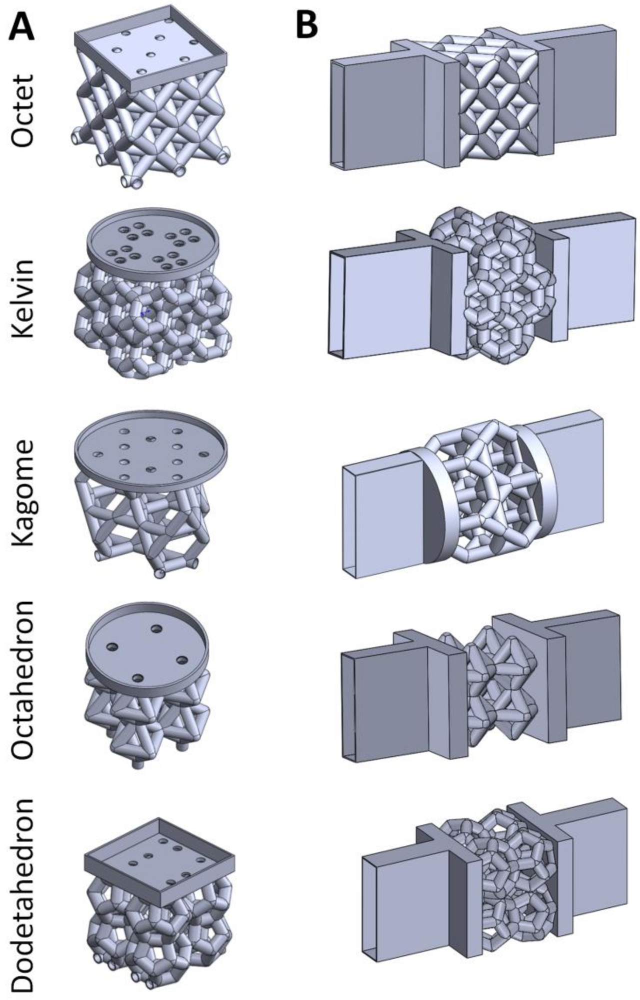

Supplementary Figure 2. Hollow scaffolds for fabricating elastomer lattices for (**A**) compressive tests and (**B**) tensile tests.

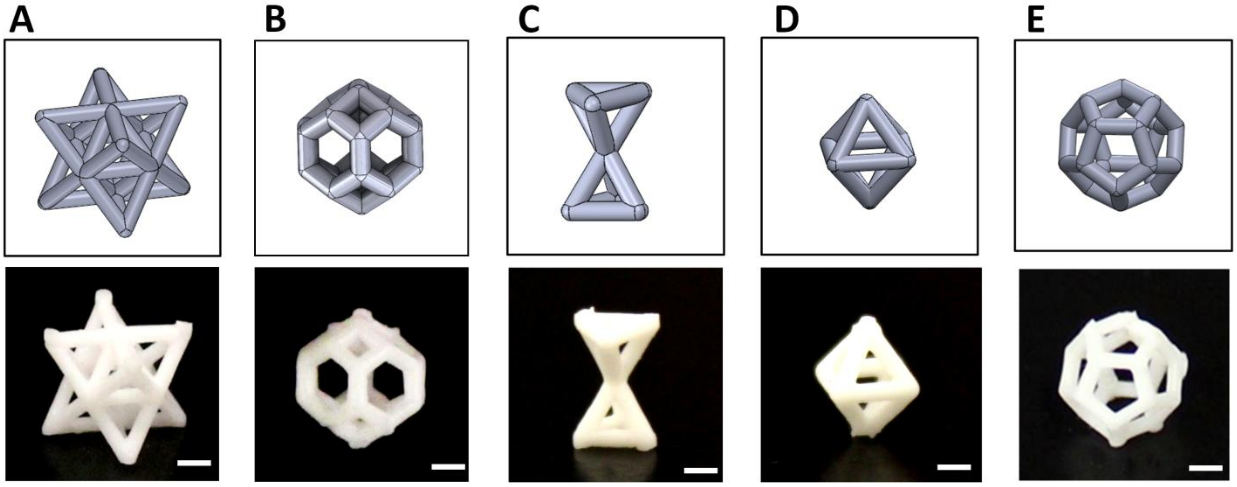

Supplementary Figure 3. CAD models and fabricated samples of unit cells of (A) Octet, (B) Kelvin, (C) Kagome, (D) Octahedron and (E) Dodecahedron lattices. All scale bars denote 1 mm.

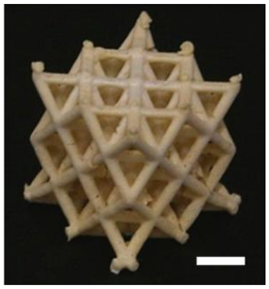

Supplementary Figure 4. Fabricated Octet lattice with Urethane elastomer. Scale bar denotes 2 mm.

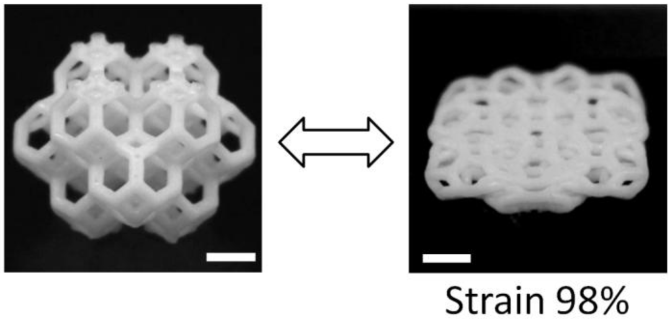

Supplementary Figure 5. A Kelvin elastomer lattice can be reversibly compressed by 98% strain. The scale bars denote 2 mm.

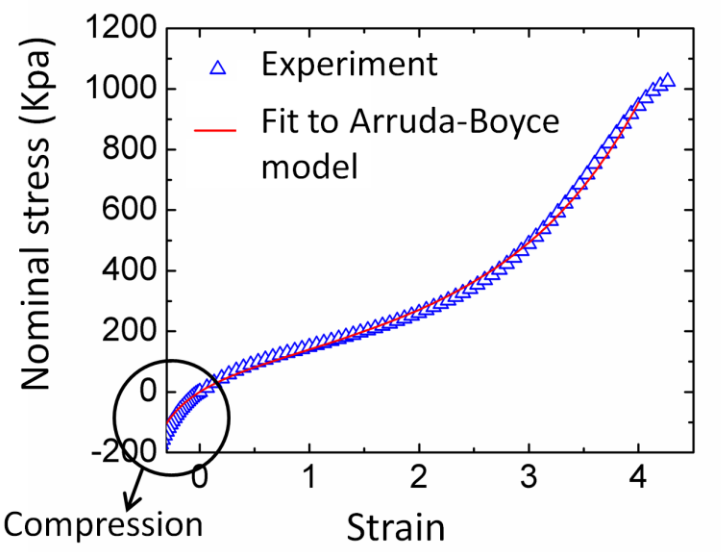

Supplementary Figure 6. Nominal stresses of the elastomer solid (Mold max NV14 A:B=10:1) under tensile and compressive strains. The compressive strains and stresses are denoted as negative. The experimental curve is fitted to Arruda-Boyce model shown in **Eq. 2** with the shear modulus of the elastomer solid as μ_0=71 kPa and λ_m=2.91.

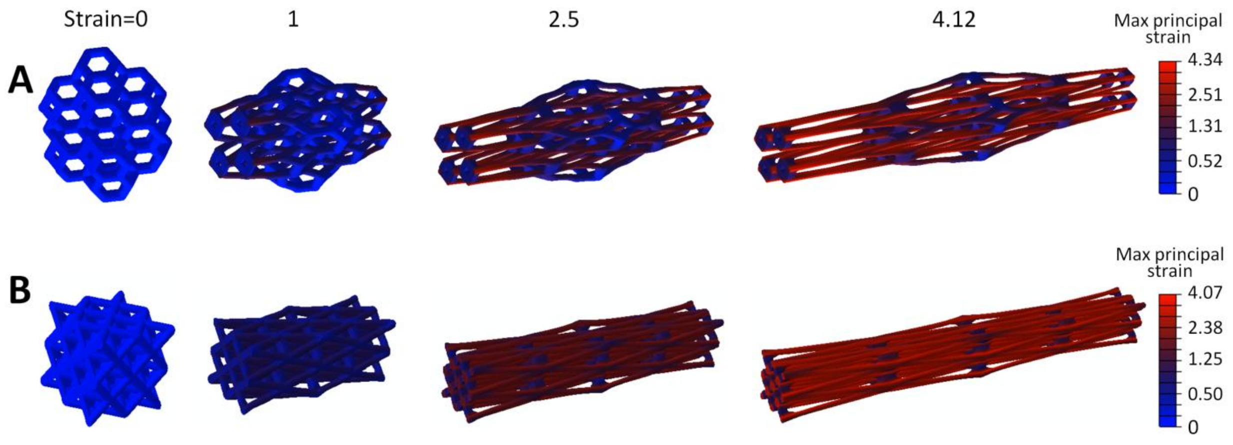

Supplementary Figure 7. Finite element simulated configurations of (**A**) Kelvin and (**B**) Octet elastomer lattices under increasing tensile strains, respectively.

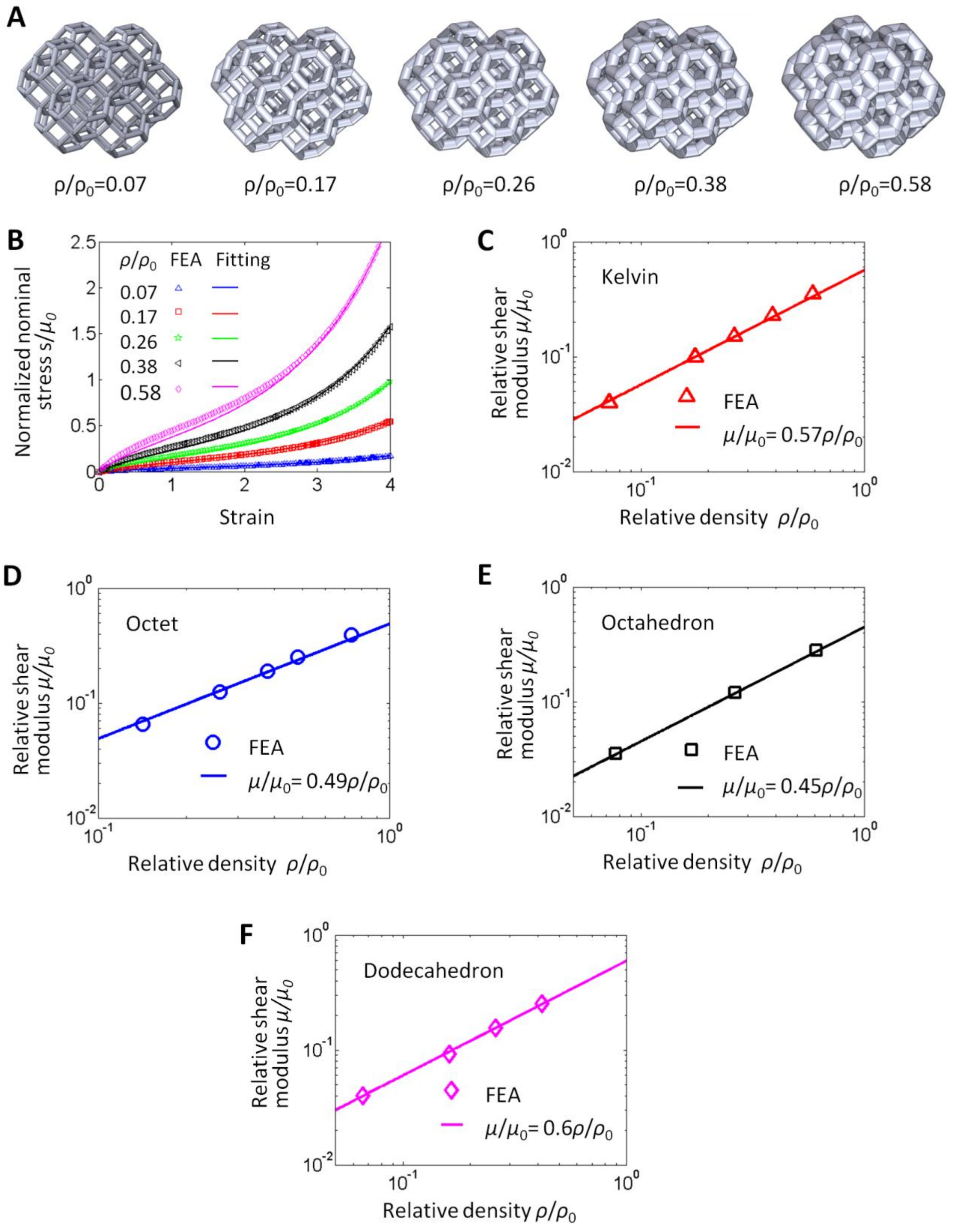

Supplementary Figure 8. (A) CAD models of Kelvin lattices with various relative densities. (**B**) Simulated and fitted nominal stresses of Kelvin elastomer lattices with various relative densities in functions of tensile strains. The Simulated stress-strain curves are fitted to the Arruda-Boyce model

shown in **Eq. 2** to obtain the effective shear modulus. **(C)** The fitted shear moduli of the Kelvin lattices plotted in a linear relationship with relative densities. With the similar method shown in (A-C), the shear moduli of (D) Octet, (E) Octahedron and (f) Dodecahedron lattices can also be calculated as linear relationships with their relative densities.

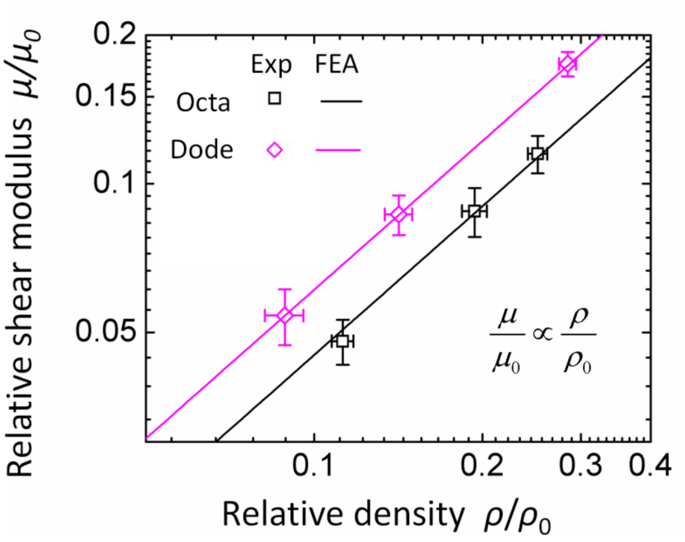

Supplementary Figure 9. Experimentally measured and finite-element simulated relative shear modulus of Octahedron and Dodecahedron elastomer lattices in functions of their relative densities.

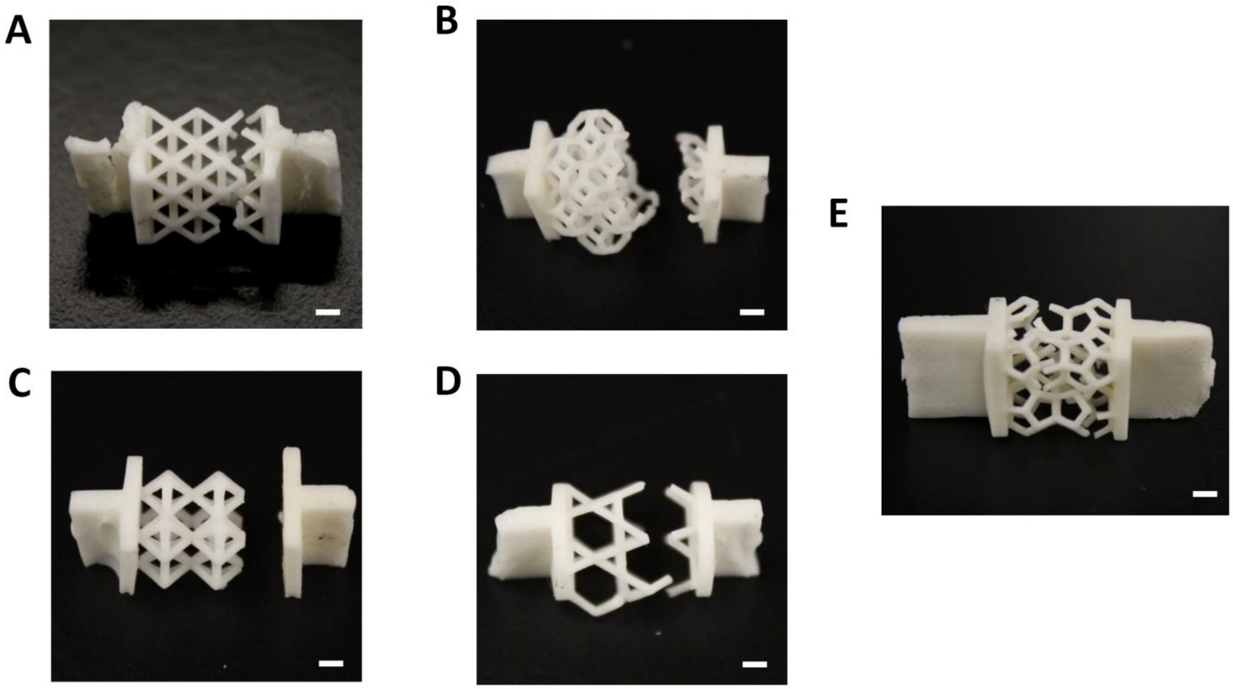

Supplementary Figure 10. Fractured samples of (**A**) Octet, (**B**) Kelvin, (**C**) Octahedron, (**D**) Kagome and (**E**) Dodecahedron lattices after extreme tensions (see example in **Supplementary Movie 1**). All scale bars denote 2 mm.

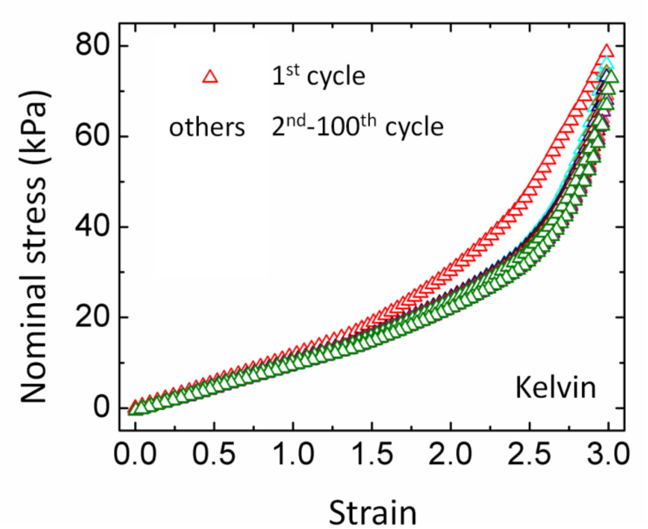

Supplementary Figure 11. Nominal stresses of a Kelvin elastomer lattice under cyclic tensile strains.

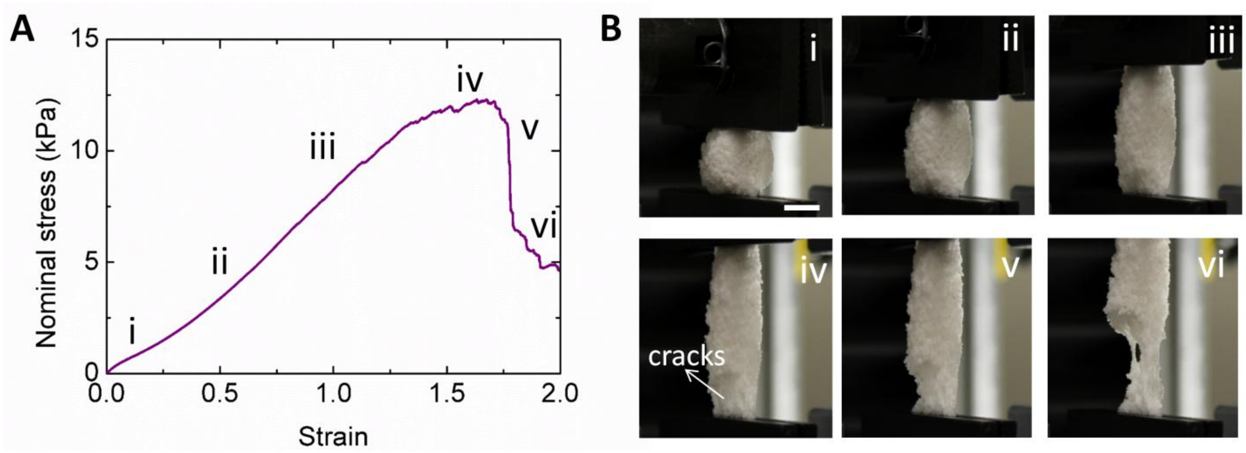

Supplementary Figure 12. (**A**) Nominal stresses of an elastomer foam in a function of the tensile strains under loading rate is 0.0167mm/s. (B) Sequential images (i-vi) of the elastomer foam under large-strain tension denoted in (A). Cracks can be easily initiated from weak beams (biii-iv). The scale bar denotes 4 mm.

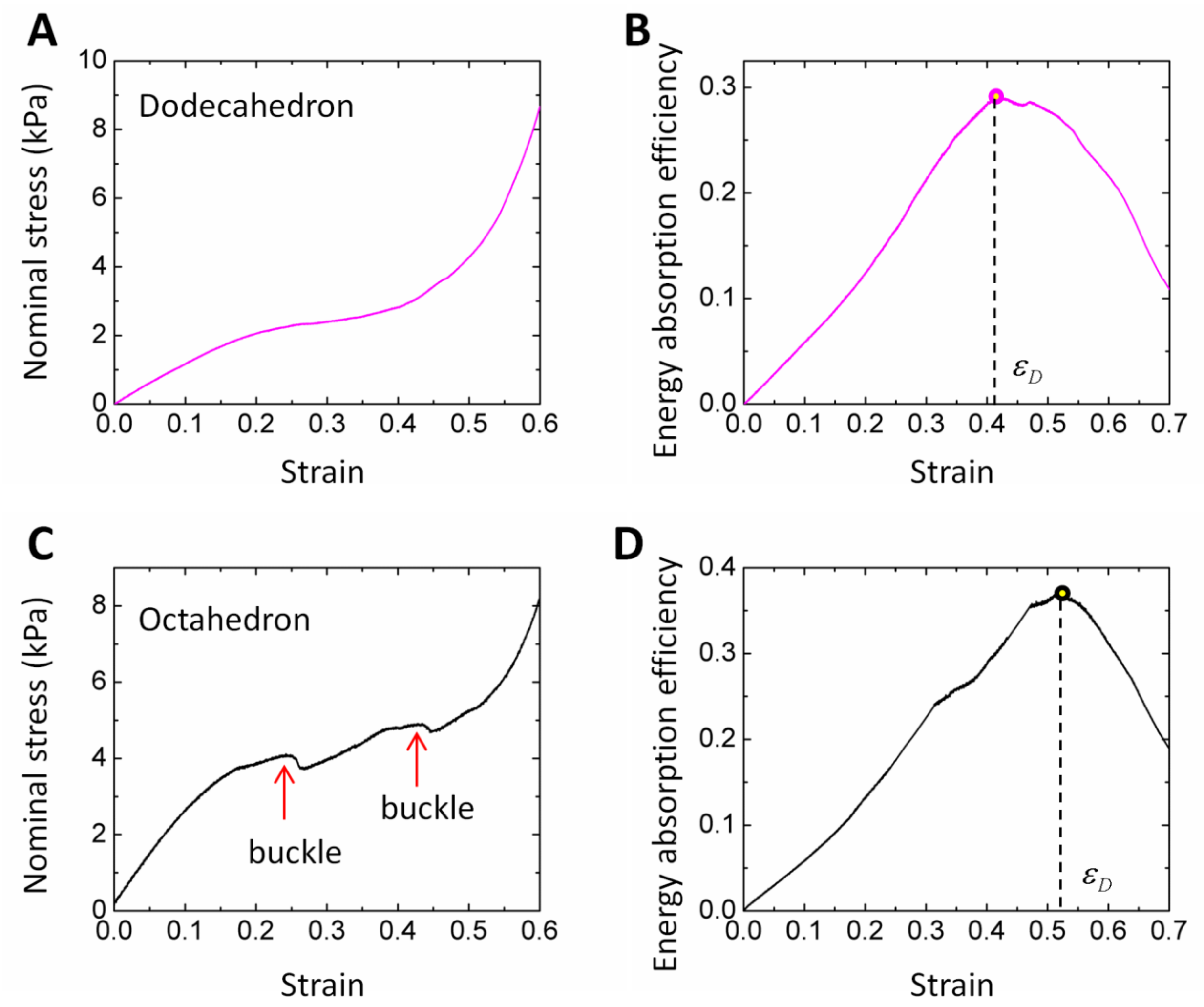

Supplementary Figure 13. (**A, C**) Nominal stresses and (**B, D**) calculated energy absorption efficiencies of (**A, B**) Dodecahedron and (**C, D**) Octahedron elastomeric lattices plotted with increasing compressive strains with loading rate 0.0167mm/s. The energy absorption efficiencies shown in **Fig. 4I** are the values marked with circles in (B) and (D). The densification strains are shown by the dash lines in (B) and (D).

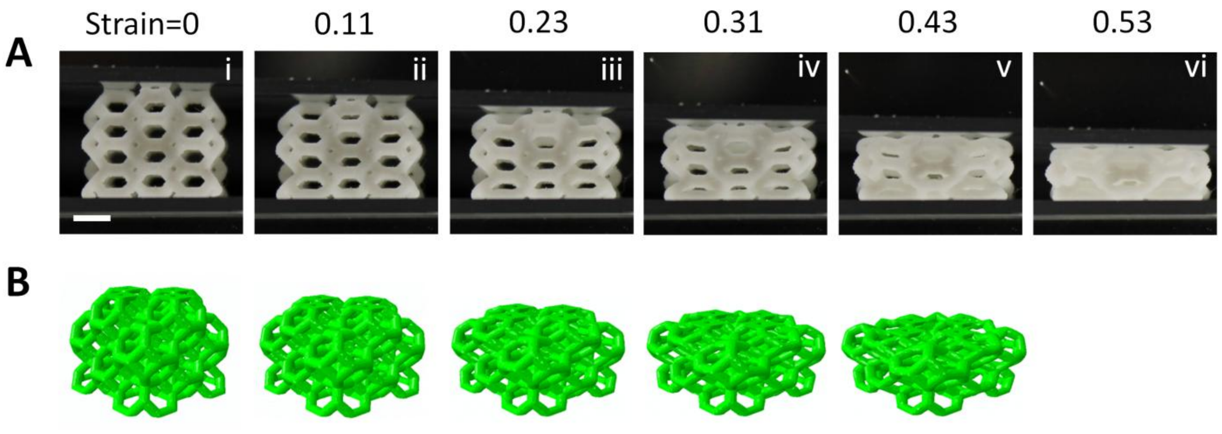

Supplementary Figure 14. Comparison of (A) experimentally observed and (B) finite-element simulated configurations of the Kelvin elastomer lattice under increasing compressive strains. The scale bar in (A) denotes 2 mm.

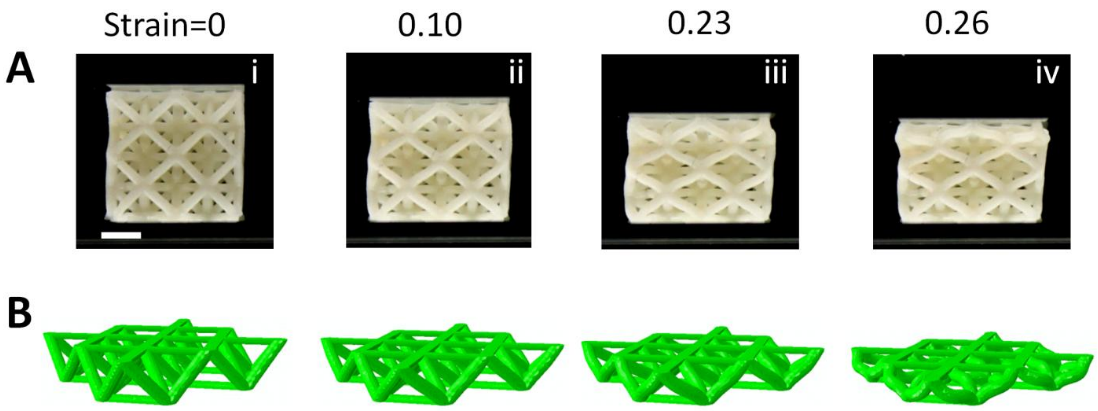

Supplementary Figure 15. Comparison of (**A**) experimentally observed and (**B**) numerically simulated configurations of the Octet elastomer lattice under increasing compressive strains. For simplicity, we only consider the top layer frame of the Octet lattice in the finite-element simulations (B). The scale bar in (A) denotes 2 mm.

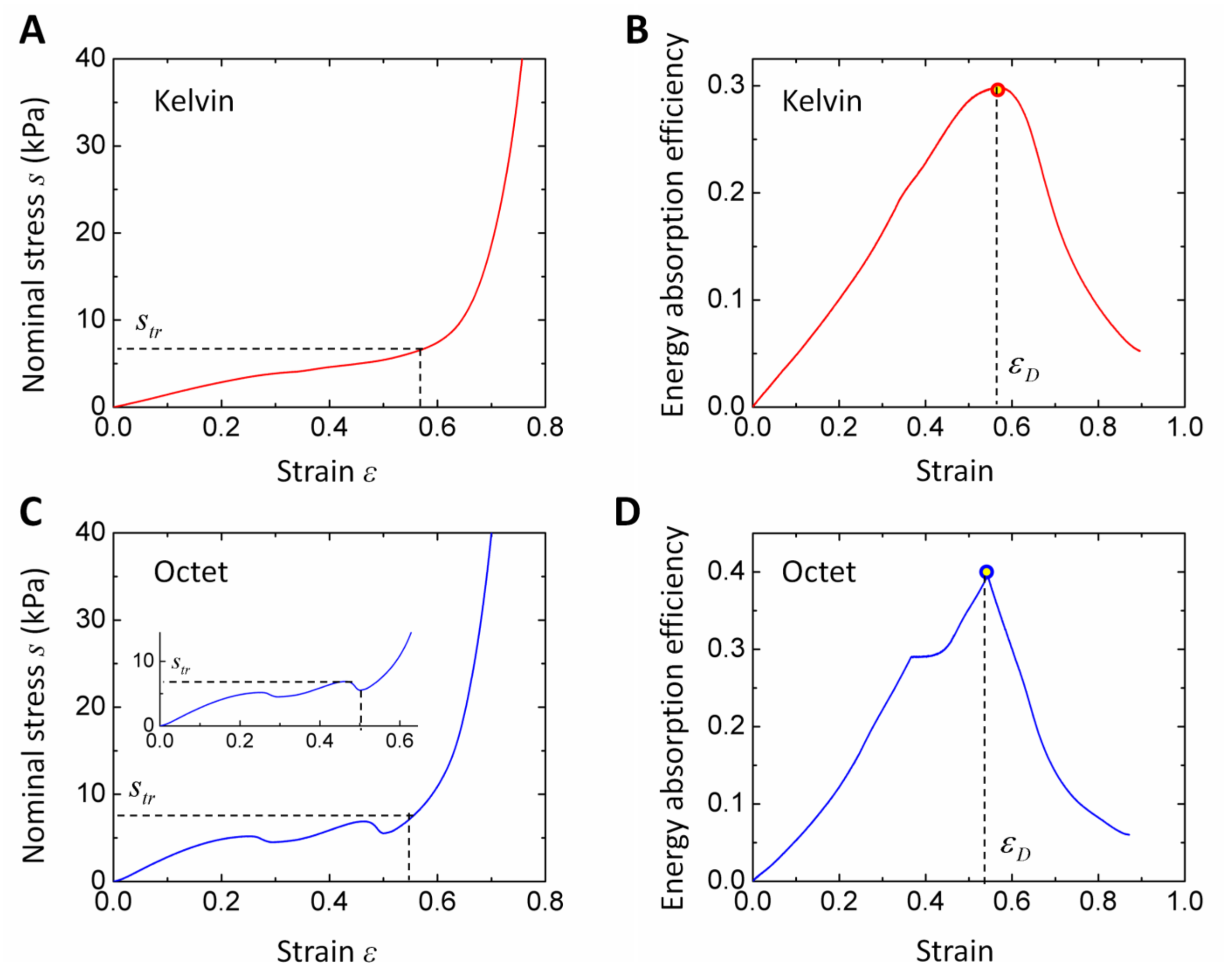

Supplementary Figure 16. (A, C) Nominal stresses and (B, D) calculated energy absorption efficiencies of (A, B) Kelvin and (C, D) Octet elastomer lattices in functions of compressive strains with loading rate 0.0167mm/s. The energy absorption efficiencies in (B) and (D) are calculated via $\eta_1 = \left(\int_0^{\varepsilon} s d\varepsilon\right) / s_{tr}$ (see Eq. S2), where ε is the increasing strain, s is the nominal stress, and $s_{tr} = \max[s(0 \rightarrow \varepsilon)]$ is the maximum transmitted stress. It is noted that s_{tr} is not necessarily equal to *s* (see a case in the inset of C). The energy absorption efficiencies shown in **Fig. 4I** are the values marked with circles in (B) and (D). The densification strains are shown by the dash lines in (B) and (D).

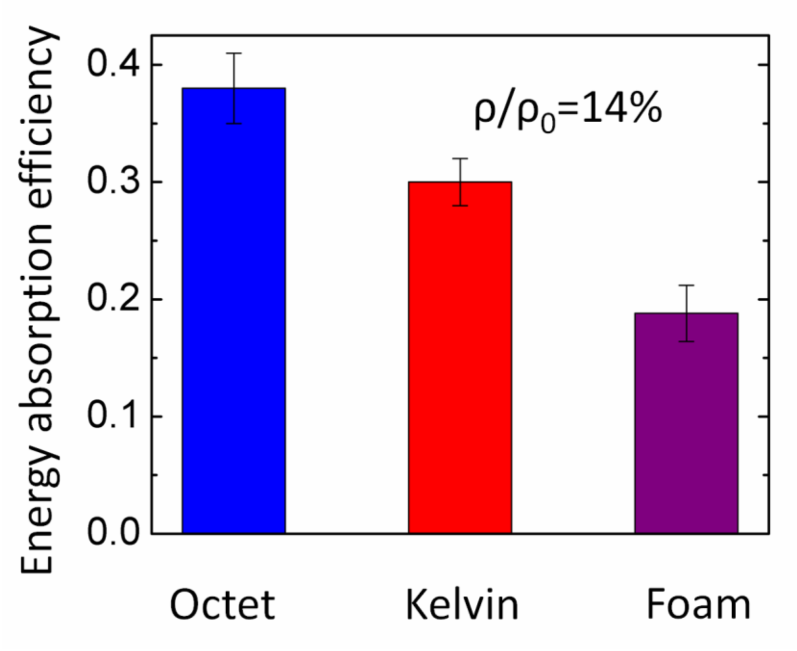

Supplementary Figure 17. Energy absorption efficiencies of Octet elastomer lattices, Kelvin elastomer lattices and elastomer foams with relative density ρ/ρ_0~14%. The loading rate is 0.0167mm/s.

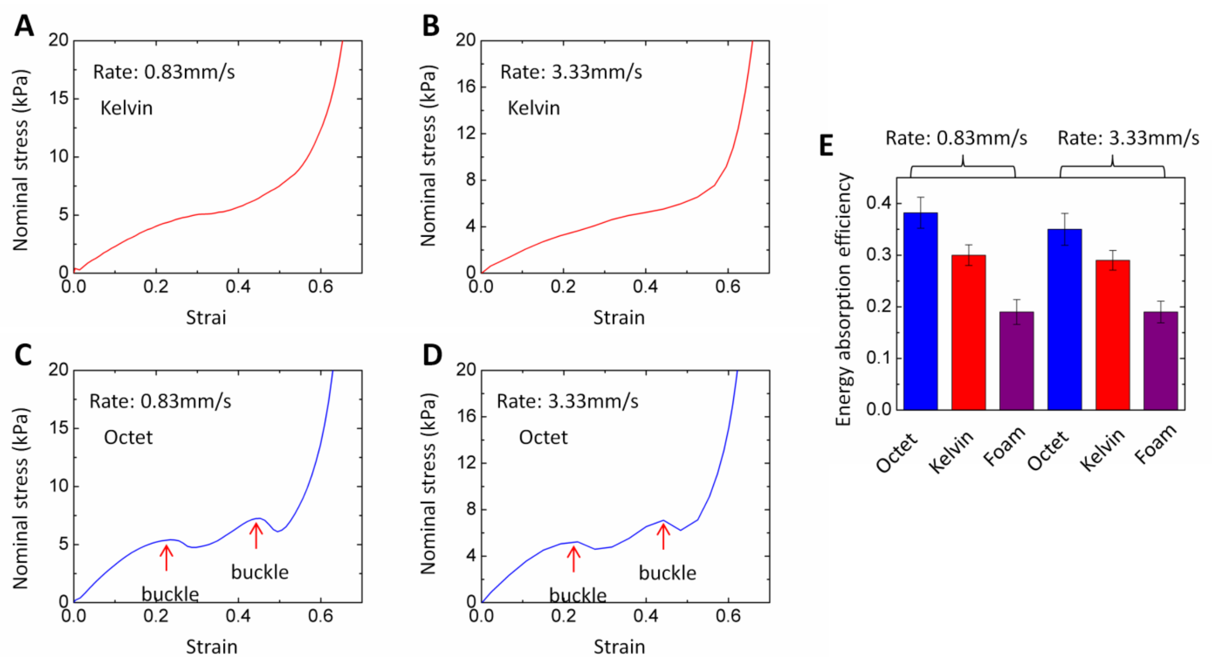

Supplementary Figure 18. Nominal stresses of (A, B) Kelvin and (C, D) Octet elastomer lattices with increasing compressive strains under loading rates (A, C) 0.83 mm/s and (B, D) 3.33 mm/s, respectively. **(E)** Energy absorption efficiencies of Octet elastomer lattices, Kelvin elastomer lattices and elastomer foams under loading rates 0.83 mm/s and 3.33 mm/s, respectively.

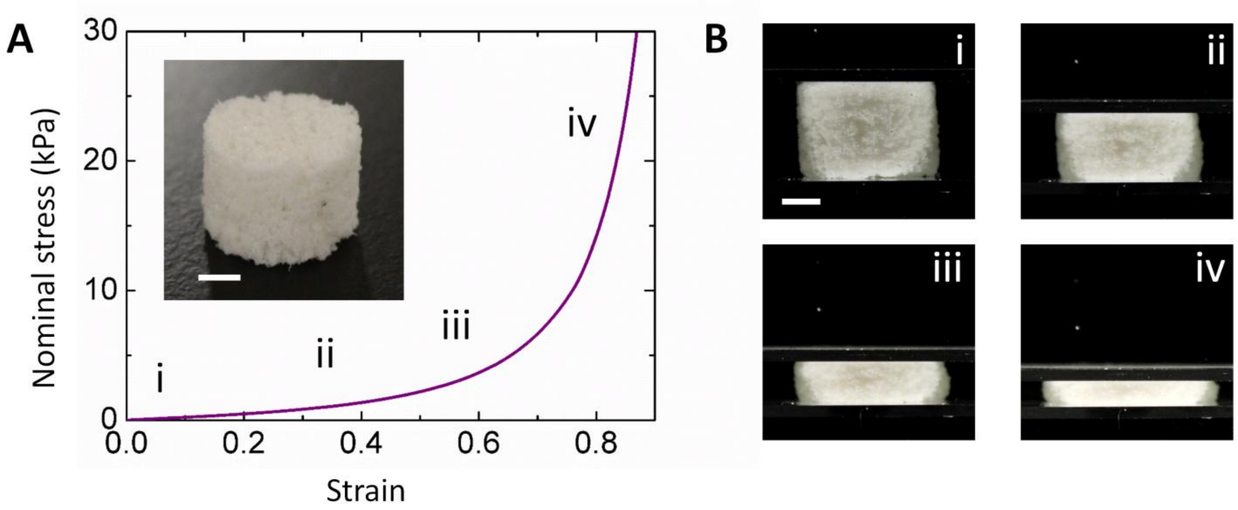

Supplementary Figure 19. (A) Nominal stresses of an elastomer foam (ρ/ρ_0~26%) in a function of the compressive strains under loading rate is 0.0167 mm/s. The inset shows the fabricated elastomer foam. **(B)** Sequential images (i-iv) of the elastomer foam under large-strain compressions denoted in (A). The scale bar denotes 2 mm.

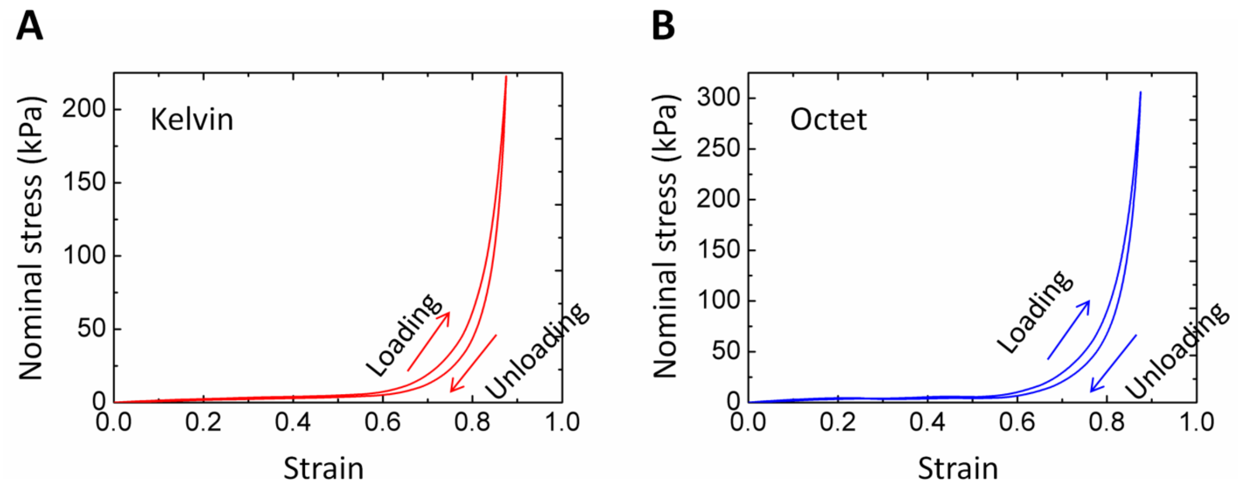

Supplementary Figure 20. Nominal stresses of (**A**) Kelvin and (**B**) Octet elastomer lattices under cyclic compressive strains.

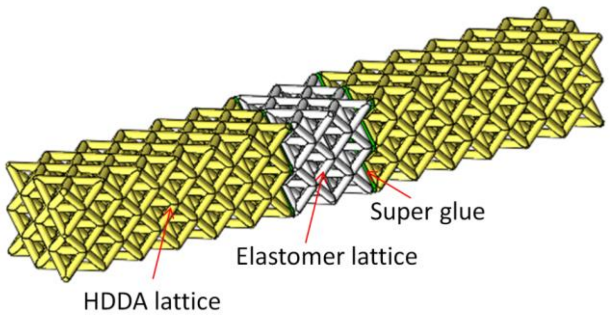

Supplementary Figure 21. Schematic to show an elastomer lattice bonded between two HDDA lattices via thin layers of super glue.

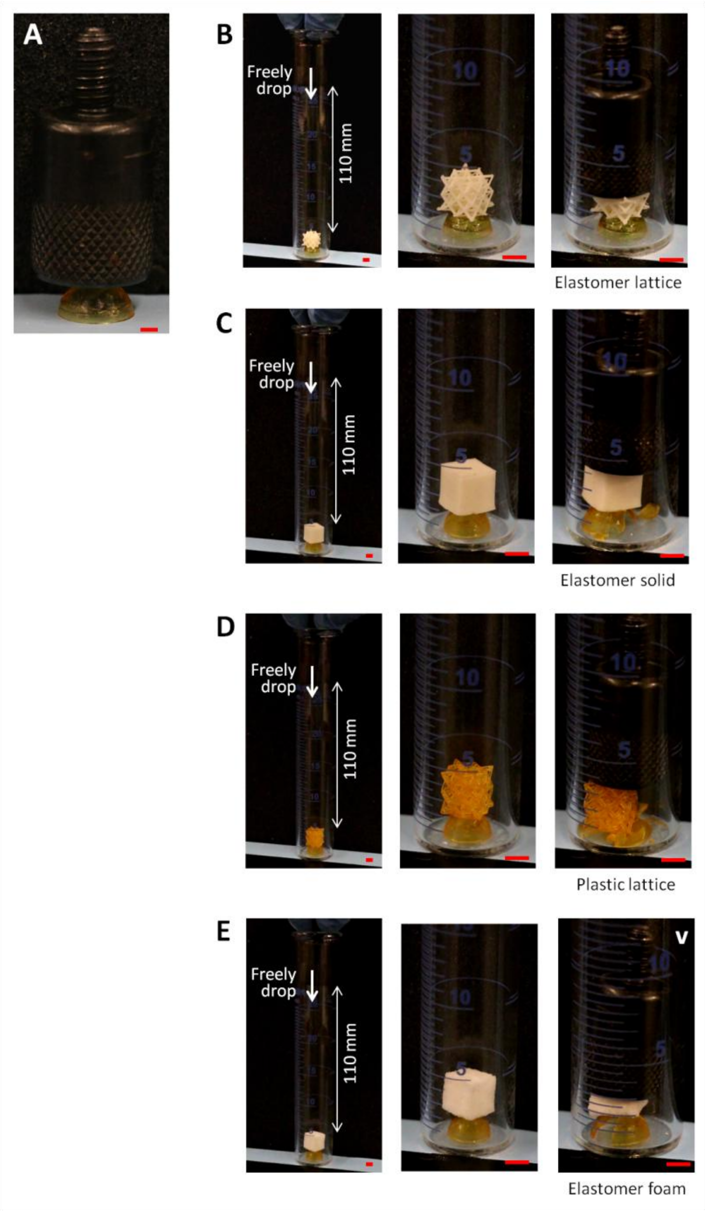

Supplementary Figure 22. (A) The fabricated plastic semi-spherical shell (0.16 g) can sustain the 36 g weight without fracture. (**B-E**) damping experiments using (**B**) elastomer lattice, (**C**) elastomer solid, (**D**) plastic lattice and (**E**) elastomer foam as dampers, respectively. All scale bars denote 2 mm.

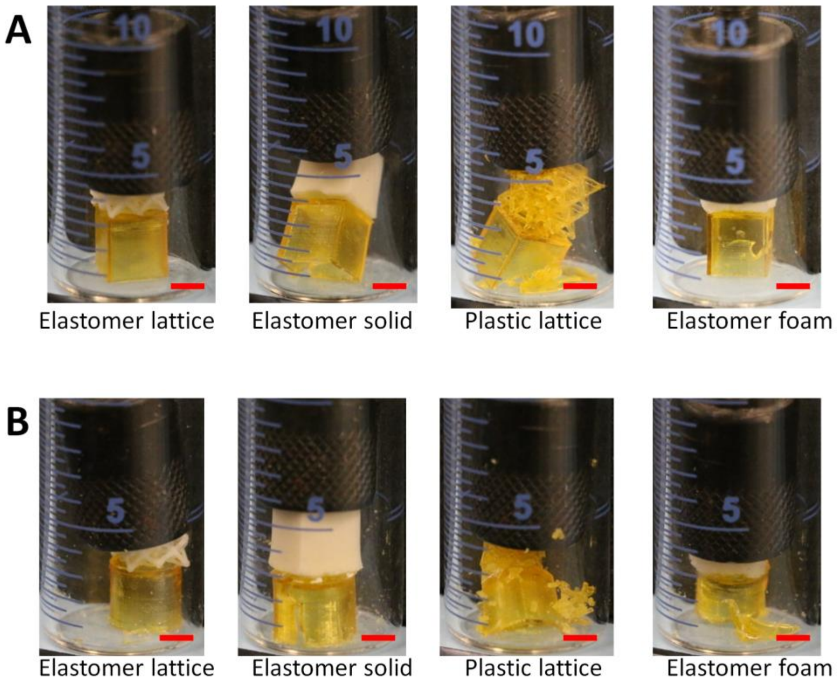

Supplementary Figure 23. Damping experiments on (**A**) cubic and (**B**) cylindrical shells using elastomer lattices, elastomer solids, plastic lattices and elastomer foams as dampers, respectively. All scale bars denote 2 mm.

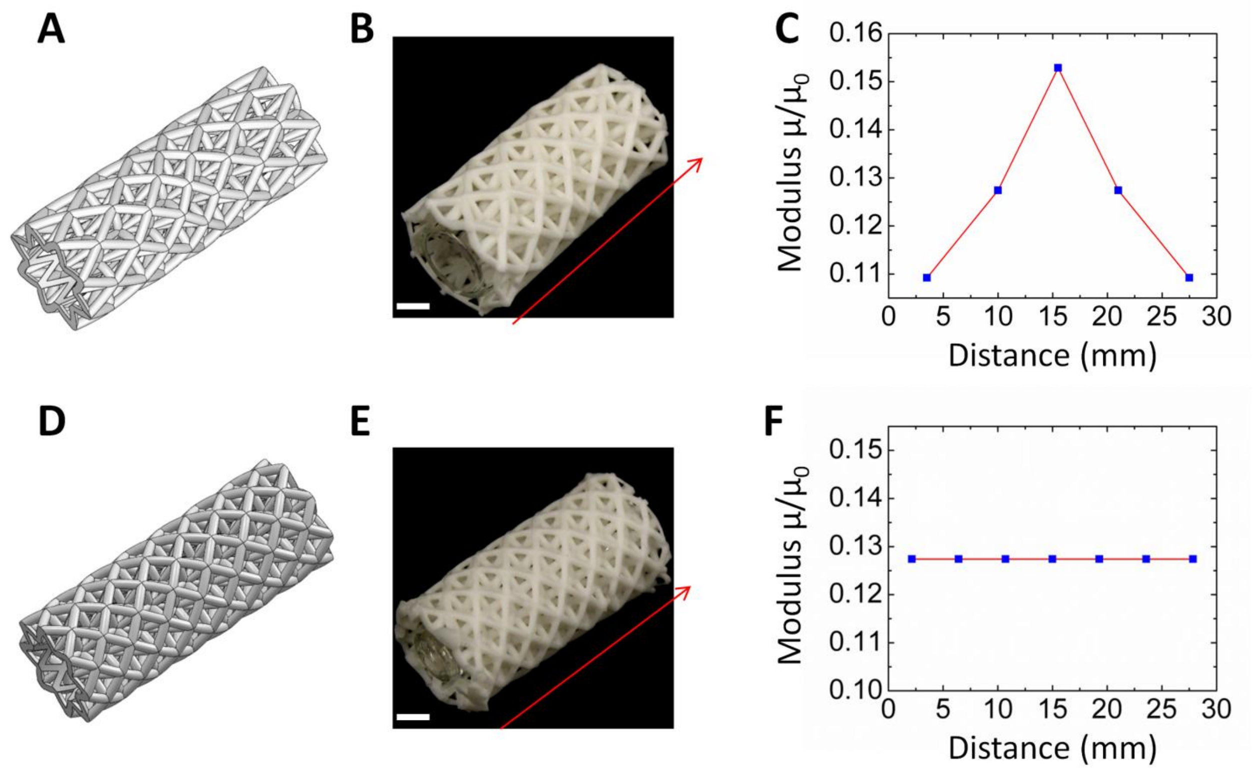

Supplementary Figure 24. (A, D) CAD models and (B, E) fabricated elastomer lattice structures with (C) gradient rigidity and (F) homogeneous rigidity, respectively. Scale bars in (B) and (E) denote 4 mm. The modulus distribution along the path marked in (B) and (E) for (C) gradient and (F) homogeneous cases, respectively. The shear modulus is calculated by $\mu/\mu_0=0.49\rho/\rho_0$.

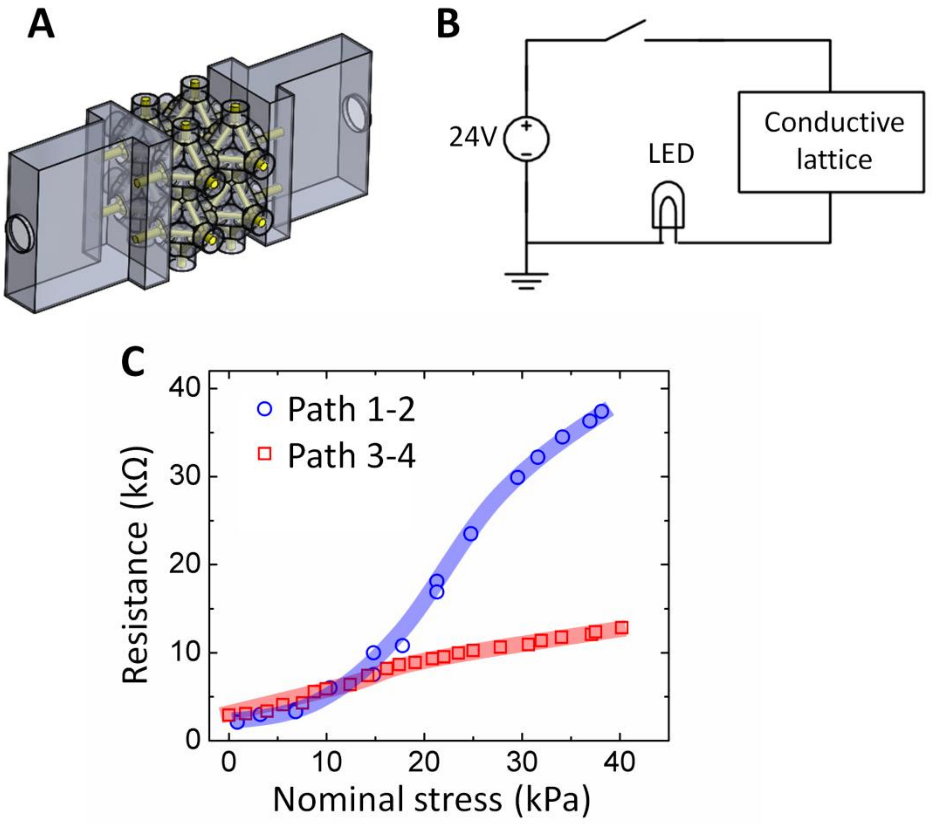

Supplementary Figure 25. (A) A hollow CAD model for fabricating a hollow Octahedron elastomer lattice. The internal yellow beams are designed to create the hollow channels within the elastomer lattices. (**B**) An electric circuit to demonstrate the conductivity of the elastomer lattice. **(C)** Resistances of path 1-2 and path 3-4 in the elastomer lattice (shown in **Fig. 5Di**) in functions of nominal stresses applied on the lattice. Using the curve in (**C**), the stretchable conductor can act as a stress/force sensor.

Supplementary movie captions

Supplementary Movie 1: A Kelvin elastomer lattice is under cyclic tensions. The strain history is from 0 increasing to 4.14, then back to 0, and increasing to 4.5. The loading and unloading rate is 0.0167 mm/s. The movie speed is 115 times of the real speed.

Supplementary Movie 2: A Kelvin elastomer lattice is under cyclic compression within strain 0-0.9. The loading and unloading rate is 0.0167 mm/s. The movie speed is 20 times of the real speed.

Supplementary Movie 3: An Octet elastomer lattice is under cyclic compression within strain 0-0.9. The loading and unloading rate is 0.0167 mm/s. The movie speed is 20 times of the real speed.

Supplementary Movie 4: A metal weight (36 g) is freely dropped from 100 mm height onto thin half-spherical HDDA shells protected by an elastomer lattice, an elastomer solid cubic, an HDDA lattice and an elastomer foam, respectively.

Supplementary Movie 5: A cylindrical elastomer-lattice structure is worn by a bending finger.

Supplementary Movie 6: An elastomer lattice with conductive channels is cyclically stretched within strain 0-2.5. The conductive path is connected to the electric circuit as shown in **Supplementary Fig. 24B** to light up an LED.

Reference

1 Evans, A. G. *et al.* Concepts for enhanced energy absorption using hollow micro-lattices. *Int. J. Impact. Eng.* **37**, 947-959 (2010).

2 Gibson, L. J. & Ashby, M. F. *Cellular solids: structure and properties*. (Cambridge university press, 1997).

3 Schaedler, T. A. *et al.* Designing metallic microlattices for energy absorber applications. *Adv. Eng. Mater.* **16**, 276-283 (2014).
